# Pseudo-cardiac tamponade owing to a large hiatal hernia

**DOI:** 10.1093/omcr/omab090

**Published:** 2021-10-26

**Authors:** Takumi Tsuchida, Yoshifumi Mizuguchi

**Affiliations:** Department of Emergency Medicine, Hokkaido University Hospital, Sapporo, Japan

A 91-year-old woman presented to our emergency department with sweating, wheezing and bloody sputum over several hours. She appeared pale, and her blood pressure was 60/31 mmHg; heart rate, 150 beats/min and oxygen saturation, 74% on room air. Physical examination revealed jugular venous distension and wheezes in both lungs. The admission electrocardiogram showed only sinus tachycardia. Transthoracic echocardiography confirmed features of cardiac tamponade and compression of the heart by a large high-echoic mass. Respiratory status improved promptly with oxygen therapy and non-invasive positive pressure ventilation, but cardiogenic shock did not improve sufficiently with extracellular fluid and noradrenaline administration. Computed tomography showed a large hiatal hernia with migration of the stomach into the mediastinum compressing the heart from the dorsal side (Fig. 1, Panel A). After decompression of the stomach via a nasogastric tube, she immediately recovered from cardiogenic shock (Fig. 1, Panel B). Although hiatal hernia with fatal complications is usually indicated for surgery, we chose conservative therapy owing to her age and poor health status. After treatment for pulmonary congestion and aspiration pneumonia, she was transferred to a rehabilitation hospital.

**Figure 1:**
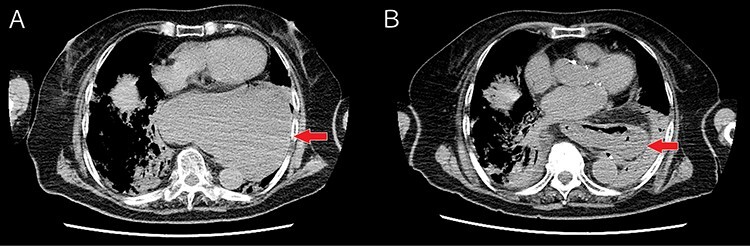
**Panel A:** Compression of the heart due to a large hiatal hernia; **Panel B:** The pressure on the heart was relieved by decompression of the stomach.

Hiatal hernia is a condition that refers to protrusion of an intraabdominal organ or organs in the mediastinum through an esophageal hiatus of the diaphragm. In most cases of hiatal hernia, the gastroesophageal junction is only mildly displaced above the diaphragm; few cases show displacement of most of the stomach into the mediastinum. Large hiatal hernia can cause a variety of arrhythmias and ST changes on the electrocardiogram [1], obstructive shock or cardiac arrest [1, 2]. Although this hemodynamic condition resembles cardiac tamponade, it differs from this condition due to the absence of pericardial effusion.

This pseudo-cardiac tamponade occurs after cardiovascular surgery [3] but might also occur in patients with unoperated hiatal hernia. In inoperable cases, dietary measures and peristaltic promoters prevent gastric distension.

## CONFLICT OF INTEREST STATEMENT

No conflicts of interest.

## FUNDING

None declared.

## ETHICAL APPROVAL

Not applicable.

## CONSENT

Written patient consent has been obtained.

## GUARANTOR

Takumi Tsuchida.

## References

[1] Krawiec K, Szczasny M, Kadej A, Piasecka M, Blaszczak P, Głowniak A. Hiatal hernia as a rare cause of cardiac complications - case based review of the literature. Ann Agric Environ Med 2021;28:20–6. 33775064 10.26444/aaem/133583

[2] Arvind A, Niec R, Hajifathalian K, Zarnegar R, Wan D. Hiatal hernia presenting with recurrent non-ST elevation myocardial infarction and cardiac tamponade. ACG Case Rep 2019;6:e00278. 10.14309/crj.0000000000000278 PMC6946202 32042843

[3] Papoulidis P, Beatty JW, Dandekar U. Hiatal hernia causing extrapericardial tamponade after coronary bypass surgery. Interact Cardiovasc Thorac Surg 2014;19:716–7. 24997185 10.1093/icvts/ivu215

