# Erratum to: Pseudo-cardiac tamponade owing to a large hiatal hernia

**DOI:** 10.1093/omcr/omab140

**Published:** 2021-12-28

**Authors:** Takumi Tsuchida, Yoshifumi Mizuguchi

**Affiliations:** 1 Department of Emergency Medicine, Hokkaido University Hospital, Sapporo, Japan; 2 Department of Cardiovascular Medicine, Hokkaido University Hospital, Sapporo, Japan

In the originally published version of this paper, the affiliation of author, Yoshifumi Mizuguchi, was incorrect. The paper has been corrected to include the correct affiliation, Department of Cardiovascular Medicine, Hokkaido University Hospital.

